# A genotype–phenotype correlation in split-hand/foot malformation type 1: further refinement of the phenotypic subregions within the 7q21.3 locus

**DOI:** 10.3389/fmolb.2023.1250714

**Published:** 2023-10-17

**Authors:** Anna Sowińska-Seidler, Magdalena Socha, Anna Szoszkiewicz, Anna Materna-Kiryluk, Aleksander Jamsheer

**Affiliations:** ^1^ Department of Medical Genetics, Poznan University of Medical Sciences, Poznan, Poland; ^2^ Institute of Molecular Biology and Biotechnology, Adam Mickiewicz University, Poznan, Poland; ^3^ Centers for Medical Genetics GENESIS, Poznan, Poland

**Keywords:** split-hand/foot malformation type 1, ectrodactyly, 7q21.3, *DLX5/6*, structural variants, *SYT1*, Baker–Gordon syndrome, whole-genome sequencing

## Abstract

**Background:** Split-hand/foot malformation type 1 (SHFM1) refers to the group of rare congenital limb disorders defined by the absence or hypoplasia of the central rays of the autopods with or without accompanying anomalies, such as hearing loss, craniofacial malformation, and ectodermal dysplasia. Consequently, the condition is characterized by clinical variability that hinders diagnostic and counseling procedures. SHFM1 is caused by pathogenic variants affecting the *DLX5/6* genes and/or their tissue-specific enhancers at the 7q21.3 locus. Herein, we report on seven patients from five unrelated Polish families affected by variable symptoms of the SHFM1 spectrum, all harboring 7q21.3 or 7q21.2-q21.3 rearrangements, and provide a genotype–phenotype correlation in the studied cohort.

**Methods:** We applied GTG banding, array-based comparative genomic hybridization (aCGH), and whole-genome sequencing (WGS) in order to identify the causative aberrations in all affected patients.

**Results:** The identified pathogenic structural variants included deletions and/or translocations involving the 7q21.3 locus, i.e., t(7;10)(q21.3;q22.2) and t(7;12)(q21.3;q21.2) in all affected individuals. Interestingly, a sporadic carrier of the latter aberration presented the SHFM1 phenotype with additional features overlapping with Baker–Gordon syndrome (BAGOS), which resulted from the translocation breakpoint at chromosome 12 within the *SYT1* gene.

**Conclusion:** Clinical variability of the studied cohort reflects the composition of the *DLX5/6* regulatory elements that were dislocated from their target genes by chromosomal rearrangements. The correlation of our data with the previously published observations enabled us to update the phenotypic subregions and regulatory units within the SHFM1 locus. In addition, we present the first case of SHFM1 and BAGOS-like phenotype that resulted from translocation breakpoints at chromosomes 7 and 12, both of which were pathogenic, and consequently, we show the first evidence that BAGOS can also result from the regulatory loss-of-function *SYT1* mutations. In this paper, we emphasize the utility of sequence-based approaches in molecular diagnostics of disorders caused by regulatory structural variants.

## 1 Introduction

Split-hand/foot malformation (SHFM), also known as ectrodactyly, is a rare congenital limb defect characterized by the median cleft of the autopod that results from hypoplasia/aplasia of the central phalanges, metacarpals, and/or metatarsals. This disorder occurs in approximately 1 in 18,000 live born infants and accounts for 8%–17% of all limb malformations (Czeizel et al., 1993). SHFM is clinically heterogeneous and presents inter- and intra-familial phenotypic variability that ranges from a mild manifestation, such as hypoplasia of a single phalanx, to the most severe form - monodactyly. Ectrodactyly may occur as an isolated malformation or in a syndromic form, which in general is less frequent but relatively common in certain types of the condition (Elliott and Evans, 2006; Gurrieri and Everman, 2013; Sowińska-Seidler et al., 2014b). There are eight known genetic loci associated with the corresponding types of the disorder, the majority of which undergo an autosomal dominant mode of inheritance with relatively frequent cases of reduced penetrance. Type 1 (SHFM1) is linked to 7q21.3 (OMIM 183600), SHFM2 is located at Xq26 (OMIM 313350), SHFM3 is associated with duplications at 10q24.3 (OMIM 246560), SHFM4 with heterozygous mutations in *TP63* (3q27) (OMIM 605289), SHFM5 with deletions at 2q31 (OMIM 606708), SHFM6 with homozygous or compound heterozygous mutations in *WNT10B* (OMIM 225300), SHFM/SHFLD3 with duplications at 17p13.3 (*BHLH9* gene) (OMIM 612576), and the recently identified EEC syndrome is associated with microdeletions at 19q13.11 or loss-of-function mutations in *UBA2* (ubiquitin-like modifier-activating enzyme 2, OMIM 613295).

The SHFM1 type of disorder, mapped to locus 7q21.3, is characterized by high clinical heterogenicity, incomplete penetrance, and sex bias, pointing to a greater penetrance in male individuals than in female individuals (for isolated SHFM only) (Elliott and Evans, 2006; Sowińska-Seidler et al., 2014b; Rasmussen et al., 2016). Syndromic forms of the condition are frequently found. Accordingly, ectrodactyly is associated with intellectual disability (ID) in 33% of cases, with craniofacial malformations (CF) in 33% of cases, and with hearing loss (HL) (SHFM1D; OMIM 220600) in 35% of cases. Some patients have ectodermal dysplasia which usually enables the diagnosis of EEC syndrome (Elliott and Evans, 2006). The SHFM1 locus encompasses five protein-coding genes, namely, *DYNC1I1*, *SLC25A13*, *SEM1*, *DLX5*, and *DLX6* (the distal-less homeobox 5/6), and a number of tissue-specific enhancers. Among the aforementioned genes, only *DLX5/6* are known to be associated with the phenotypic manifestation of the disorder, which has been functionally demonstrated in mouse model studies. *DLX5/6* form a digenic cluster, a part of a *Dlx* homeobox transcription factor family, and are essential in promoting osteogenesis. Both genes upregulate chondrocyte and osteoblast differentiation (Samee et al., 2008). The homozygous double knockout of both genes in mice (*Dlx5/Dlx6* ^*−/−*^) results in the SHFM1 phenotype derived from the genes’ loss-of-function in the apical ectodermal ridge (AER) of the limb buds or in the otic vesicle (Acampora et al., 1999; Merlo et al., 2002a; 2002b; Robledo et al., 2002; Conte et al., 2016). The histological analysis revealed an impairment of the endochondral ossification of bones of the appendicular and axial skeleton in the mutants (Robledo et al., 2002). Additional findings that support these observations came with reports of SHFM1 patients harboring homozygous or heterozygous loss-of-function variants in the *DLX5* gene (Shamseldin et al., 2012; Sowińska-Seidler et al., 2014a; Wang et al., 2014) and a single report of the heterozygous missense variant in *DLX6* (Ullah et al., 2017). In humans, however, mutations that directly affect the gene sequence, point mutations or intragenic deletions, are not the most frequent pathogenic events. In fact, in the majority of SHFM1 cases, the disorder is related to a “position effect” which is associated with structural variants (SVs), i.e., deletions, translocations, inversions, and duplications, that disrupt the *DLX5/6* regulatory environment of the genes’ topologically associated domain (TAD). There are at least 16 elements that play an established role in the regulation of tissue-specific *Dlx5/6* expression, as shown in mouse and zebrafish enhancer assays, including the limb-, brain-, branchial arch-, otic vesicle-, and heart-specific enhancers (Zerucha et al., 2000; Pennacchio et al., 2006; Verzi et al., 2007; Kouwenhoven et al., 2010; Birnbaum et al., 2012). It has been shown that the clinical manifestation of the disorder in SHFM1 patients harboring pathogenic SVs depends on the genomic location of the variant’s breakpoints and, consequently, on the type and number of the tissue-specific *DLX5/6* enhancers that have been dislocated from their target genes (reviewed in Birnbaum et al. (2012); Rasmussen et al. (2016)). Accordingly, the SHFM1 locus has been organized into three phenotypic subregions associated with: 1) isolated SHFM, 2) SHFM and HL, and 3) SHFM, HL, and CF (proposed by Rasmussen et al. (2016)). Nevertheless, the critical region for the SHFM1 phenotypic manifestation maps to a 103 kb segment, which encompasses two enhancers (eExon15 and eExon17) located within exons 15 and 17 of the *DYNC1I1* gene, respectively, and therefore overlaps with subregion 1 for isolated SHFM (Tayebi et al., 2014).

Herein, we report on seven patients from five unrelated Polish families affected by SHFM1 and discuss two previously described families from our SHFM1 cohort (Tayebi et al., 2014). In all examined individuals, the disorder resulted from chromosomal rearrangements at the 7q21.3 locus, i.e., deletions and reciprocal translocations. The cohort was clinically heterogeneous since the patients presented with a variety of symptoms ranging from isolated ectrodactyly to EEC syndrome. Interestingly, in addition to the typical SHFM1 manifestation, one sporadic case, harboring a reciprocal translocation t(7;12)(q21.3;q21.2), presented features overlapping with Baker–Gordon syndrome (BAGOS) (OMIM 618218). This striking phenotype was associated with the translocation breakpoint mapped within the sequence of the *SYT1* gene, which resulted in a gene loss-of-function mutation. Until now, SVs affecting *SYT1* have never been reported to cause BAGOS, which highlights the novelty of our finding. In addition to conventional diagnostic assays that enabled the identification of SVs, we applied the whole-genome sequencing (WGS) approach in order to determine the breakpoints of the balanced translocations and search for a second pathogenic variant in one of the families with incomplete penetrance of the trait. In this paper, we provide a genotype–phenotype correlation of the patients from our cohort and discuss it in relation to the previously proposed phenotypic subregions within the SHFM1 locus.

## 2 Materials and methods

### 2.1 Split-hand/foot malformation type 1 families

Five Polish families were referred for genetic consultation due to limb anomalies characterized by a spectrum of split-hand/foot malformation. Genomic DNA of all probands and their examined family members was extracted from peripheral blood lymphocytes using standard protocols. All patients agreed to participate in this study, and written informed consent was obtained from all individuals or their legal guardians prior to genetic testing. The Institutional Review Board of the Poznan University of Medical Sciences approved this study.

### 2.2 Karyotyping and microarray-based approaches

#### 2.2.1 Karyotyping

Karyotyping was performed on peripheral blood lymphocytes of the index patient from family 4, her children (P4.1, P4.2, and P4.3, respectively), and patient P5. We used conventional GTG banding at 550 band resolution per haploid genome.

#### 2.2.2 Array comparative genomic hybridization

Various formats of Agilent™ (Agilent Technologies, Santa Clara, CA) array comparative genomic hybridization (aCGH) were used to detect copy number variants (CNVs) in the index patients from families 1–4 - format 180K: patients P1 and P3; format 60K: patient P2; and format 1M: patient P4.1. The assay was performed according to the standard protocols provided by the manufacturers, using a commercial (Agilent™) or non-commercial male or female control DNA as a reference for hybridization approaches. The results were analyzed using Agilent CytoGenomics 5.0.2.5 software based on the ADM2 segmentation algorithm in order to identify CNVs. We applied thresholds of 0.4 for gains and −0.4 for losses and a filter of three neighboring probes to indicate the aberration (threshold: 6.0; window size: 0.2 Mb).

### 2.3 Real-time quantitative PCR

In order to evaluate CNVs identified with aCGH and to narrow down the deletion regions, we applied a real-time quantitative PCR (qPCR) assay using a set of primer pairs located within the SHFM1 locus. The primers were designed using Primer3Plus version 3.3.0 software^1^ and validated using the SNPCheck v3^2^ and Ensembl BLAST/BLAT^3^ online tools. The copy number in each of the target regions was estimated using the comparative ΔΔCt method and non-commercial control DNA as a calibrator. The target sequences were normalized to albumin (*ALB*). We used the primer pair designed for factor VIII (*F8*) located on the X chromosome in order to ensure the reliability of the assay. The reactions were carried out on the ViiA™ 7 Real-Time thermal cycler using Power SYBR™ Green PCR Master Mix on a 384-well setup in a total volume of 12 μl and analyzed using ViiA™ 7 software (Applied Biosystems). All reactions were run in triplicate. The reaction conditions are available upon request. For primer sequences, see Supplementary Table S1.

### 2.4 Whole-genome sequencing

Whole-genome sequencing was performed in order to determine the exact breakpoints of the reciprocal translocations in index patients from families 4 and 5 and search for the genetic modifiers that contributed to the variable phenotypic expression of the disorder between individuals from family 4 - the mother (P4.1) and her daughter (P4.3). The sequencing library preparation was performed by Macrogen Inc. (Seoul, South Korea) using TruSeq DNA PCR-Free kits (Illumina Inc., San Diego, California, United States) and 550 bp inserts. The samples were subsequently paired-end-sequenced using 150 bp reads on the Illumina NovaSeq 6000 platform following standard protocols. Bioinformatic analyses were performed as reported previously (Sowińska-Seidler et al., 2020). Additionally, structural variants were called and genotyped using smoove v.0.2.6^4^. The comparative analysis between patients P4.1 and P4.3 was performed using two sets of data, namely, variants unique for the mother (P4.1) and absent in the daughter (P4.3) (search for putative modifiers that contributed to a complete manifestation of the disorder - the ectrodactyly) and, reversely, the collection of variants present in the daughter and absent in the mother (search for putative modifiers that enabled the phenotypic rescue and contributed to the manifestation of a milder phenotype - the brachydactyly). The variants were prioritized using the minor allele frequency threshold of below or equal to 3% and an in-house panel encompassing 402 genes related to skeletal disorders, as well as a panel of downstream targets of *DLX5/6* and upstream regulators of both genes. The pathogenicity of selected variants was evaluated according to ACMG criteria using the VarSome^5^ online prediction tool.

### 2.5 Breakpoint sequencing

The exact breakpoints of the deletions identified in patients P1, P2, and P3 were determined using PCR, followed by Sanger sequencing with primers designed to amplify the DNA fragment flanking the deleted region from its 5′- and 3′-ends. In addition, PCR–Sanger sequencing was used to confirm the translocation breakpoints established previously by WGS in patients P4.1, P4.2, P4.3, and P5. We used Ranger Mix to run all PCR reactions (Bioline Reagents Ltd.). PCR products were sequenced using dye-terminator chemistry (kit v.3, ABI 3130xl) and run on an automated sequencer, Applied Biosystems PRISM 3700 DNA analyzer. The reaction conditions are available upon request. The primers were designed using Primer3Plus version 3.3.0 software^1^ and validated using the SNPCheck v3^2^ and Ensembl BLAST/BLAT^3^ online tools. For primer sequences, see Supplementary Table S2.

### 2.6 *SYT1* relative expression in PBMCs of patient P5

Total RNA was extracted from peripheral blood mononuclear cells (PBMCs) derived from patient P5 and healthy controls by means of the RNeasy Mini Kit (QIAGEN, #74106) according to the protocol provided by the manufacturer. PBMCs were separated from whole blood in a density gradient of Histopaque-1077 (Sigma-Aldrich). A measure of 1 mg of total RNA was subjected to DNase I treatment (Thermo Scientific™ EN0525) and subsequently reverse-transcribed into cDNA using the RevertAid First Strand cDNA Synthesis Kit and random hexamer primer (Thermo Scientific™). cDNA-specific primers were designed using the online tool PrimerBLAST^6^. The relative expression level of *SYT1* was analyzed by means of the comparative ΔΔCt method to establish the expression fold change between the patient and controls. We used a *TBP* housekeeping gene as a reference for normalization. The reactions were carried out on the ViiA™ 7 Real-Time thermal cycler using Power SYBR™ Green PCR Master Mix (Applied Biosystems) on a 384-well setup in a total volume of 12 μl. All reactions were run in triplicate. Statistical significance was calculated via the one-tailed single-sample Z-test. The reaction conditions are available upon request. For primer sequences, see Supplementary Table S3.

## 3 Results

### 3.1 Clinical report

#### 3.1.1 Family 1 (sporadic)

**FIGURE 1:**
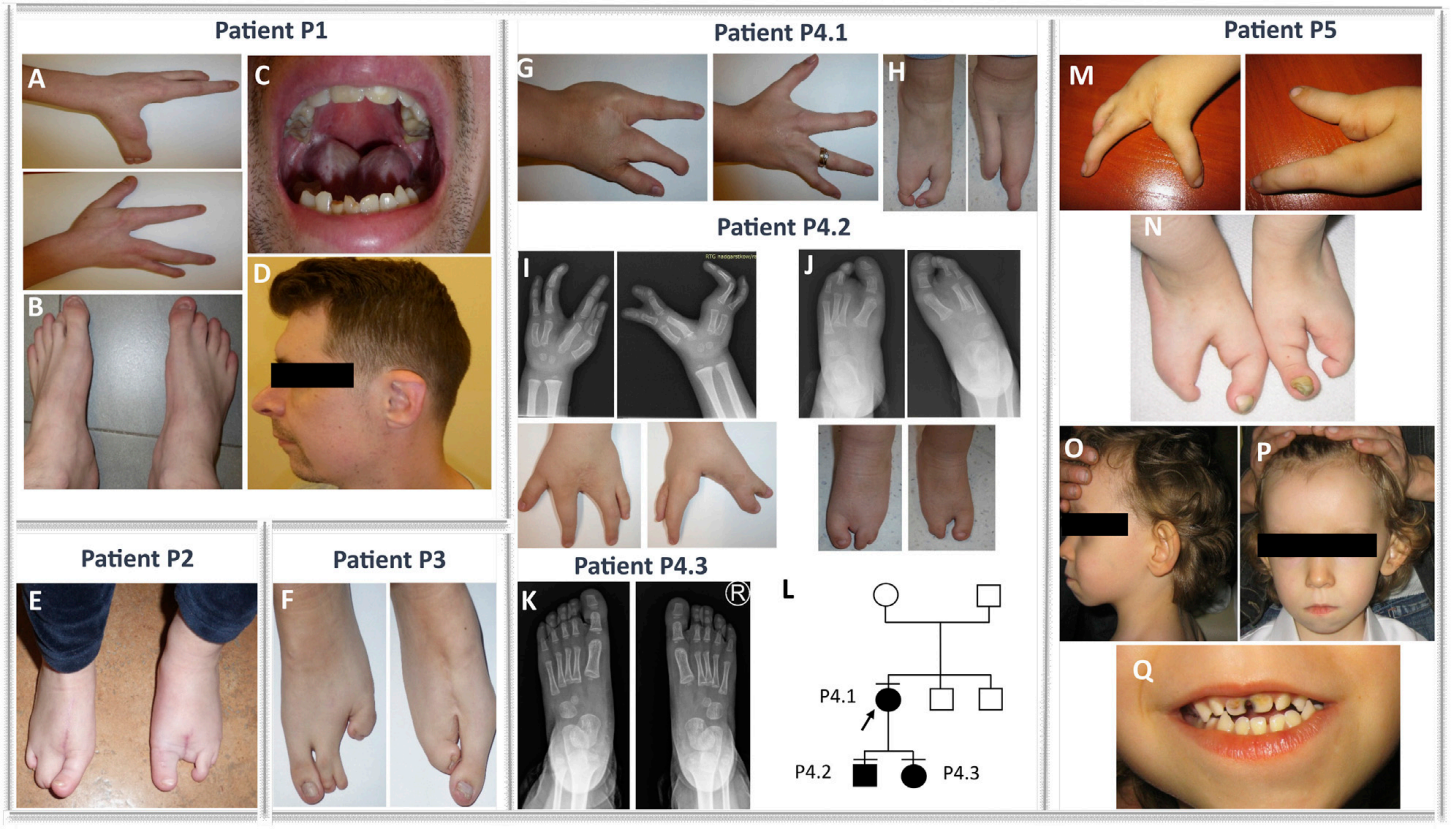
Phenotypes of families 1, 2, 3, 4, and 5. Bilateral ectrodactyly of hands **(A)** and feet **(B)**, enamel and nail hypoplasia **(A–C)**, a tendency for tooth decay **(C)**, and facial dysmorphism, e.g., micrognathia, retrognathia, and overfolded helix **(D)** in the patient from family 1 (P1). Isolated bilateral ectrodactyly of feet **(E)** of the index family 2 (P2). Isolated bilateral ectrodactyly of feet composed of hypoplasia and syndactyly of the second and third and the fourth and fifth toe of the right foot, with the apparent absence of middle toes of the left foot with clinodactyly and broadening of the hallux, and syndactyly of the fourth and fifth toes **(F)** in the proband from family 3 (P3). Bilateral isolated ectrodactyly of hands **(G)** and feet **(H)** of the index patient from family 4 (P4.1) and bilateral ectrodactyly of all four limbs **(I–J)** in her son (P4.2). A milder phenotype (slight shortening of the second and third toe of both feet) **(K)** is presented in the daughter of the index (P4.3). **(L)** Pedigree of family 4. Bilateral ectrodactyly of hands **(M)** and feet **(N)** with the absence of central fingers and toes, facial dysmorphism, i.e., micrognathia, low-set posteriorly rotated and protruding ears **(O)**, smooth philtrum, prominent high forehead with V-shaped hairline **(P)**, thin upper lip, tooth decay **(Q)**, and enamel and nail hypoplasia **(M, N, Q)** in the patient from family 5 (P5).

The proband from family 1 (P1) was a sporadic male patient of Polish ethnicity (referred for a genetic consultation at the age of 33 years). He was conceived by a healthy non-consanguineous couple as their second child. The patient was diagnosed with EEC syndrome due to bilateral ectrodactyly of hands and feet, enamel and nail hypoplasia, and a tendency for tooth decay (Figures 1A–C). In addition, the proband was affected with hearing loss, discrete facial dysmorphism (e.g., micrognathia and retrognathia) (Figure 1C), and an overfolded helix (Figure 1D). Intellectual development was normal.

#### 3.1.2 Family 2 (sporadic)

The proband from family 2 (P2) was a 27-year-old sporadic female patient of Polish ethnicity. She was conceived by a healthy, non-consanguineous couple as their first child. The patient was diagnosed with an isolated bilateral ectrodactyly of the feet composed of hypoplasia and syndactyly of the second and third and the fourth and fifth toes of the right foot and the absence of central toes of the left foot with clinodactyly and broadening of the hallux and syndactyly of the fourth and fifth toes (Figure 1E). The hands were normal. Intellectual development was normal.

#### 3.1.3 Family 3 (sporadic)

The index from family 3 (P3) was a 3-year-old female sporadic patient of Polish ethnicity. She was conceived by a healthy, non-consanguineous couple as their second child. The patient was diagnosed with isolated bilateral ectrodactyly of the feet with unaffected hands (Figure 1F). Intellectual development was normal.

#### 3.1.4 Family 4 (familial)

The proband (P4.1) was a female individual of Polish origin. She was conceived by a healthy, non-consanguineous couple as their first child. She visited a genetic clinic at the age of 24 years. The index was affected with bilateral isolated ectrodactyly of hands and feet (Figures 1G,H). Her son (P4.2) was referred for a genetic consultation in the clinic at the age of 4 years and was diagnosed with the same condition as his mother - bilateral SHFM (Figures 1I,J). Next, we examined the 2-year-old daughter (P4.3) of the index patient. The girl was affected with slight shortening of the second and third toe of both feet (Figure 1K). Intellectual development of all patients presenting with ectrodactyly was normal. The pedigree of family 4 is shown in Figure 1L.

#### 3.1.5 Family 5 (sporadic)

The index (P5) was a sporadic male patient of Polish ethnicity. He was conceived by a healthy, non-consanguineous couple and referred for genetic consultation at the age of 4 years. The mother recalls that she was exposed to industrial chemical cleaning products during pregnancy with the index patient. The proband was affected with EEC syndrome and additional symptoms overlapping with BAGOS. Upon clinical examination, we found bilateral ectrodactyly of hands and feet with the absence of central fingers and toes (Figures 1M,N), tooth decay, enamel and nail hypoplasia (Figures 1N,Q), and facial dysmorphism, i.e., micrognathia, smooth philtrum, thin upper lip, almond-shaped eyes, prominent high forehead with V-shaped hairline, low-set posteriorly rotated and protruding ears, strabismus (Figures 1O–Q), cryptorchidism, atrial septal defect, global developmental delay, absent speech, and hearing loss.

Bone fractures, osteopenia, and osteoporosis were not reported in any of the examined patients. The phenotypic features of all examined patients are summarized in Table 1.

**TABLE 1:** Molecular and clinical summary of patients harboring aberrations at the 7q21.2–q21.3 locus from the Polish cohort.

*ID*	*Aberration*	*Size*	Deleted gene/enhancer**											*Phenotype*
			hs1626 and hs1831	eDLX#26	eDLX#24	eExon15, eExon17, and eDLX#23	hs1642	eDlx#19 and eDlx#18	eDlx#14 and eDLX#16	BS1	eDlx#8 and 5 K-Del	eDlx#4, I56i, and I56ii	*DLX5* and *DLX6*	
***P1***	seq[GR38] del(7)(q21.2q21.3) NC_000007.14:g.93032717_97617672del	4.6 Mb	+	+	+	+	+	+	+	+	+	+	+	EEC syndrome: bilateral ectrodactyly of hands and feet, enamel and nail hypoplasia, tendency for tooth decay, hearing loss, micrognathia, retrognathia, and overfolded helix
***P2***	seq[GRCh38] del(7)(q21.3) NC_000007.14:g.95979917_96148982del	169 kb	-	-	+	+	-	-	-	-	-	-	-	Ectrodactyly of feet
***P3***	seq[GRCh38] del(7)(q21.3) NC_000007.14:g. 96035039_96181419del	146 kb	-	-	+	+	-	-	-	-	-	-	-	Ectrodactyly of feet
F6*	arr[GRCh38] 7q21.3 (96064029_96233057)x1	167 kb	-	-	+	+	-	-	-	-	-	-	-	Ectrodactyly of hands and feet (classic SHFM)
F7*	seq[GR38] del(7)(q21.3) NC_000007.14:g.96037734_96242732del	205 kb	-	-	+	+	-	-	-	-	-	-	-	Ectrodactyly of hands and feet (variable expression, ranging from severe cleft in four limbs to hypoplasia of the central digit of one hand, and mild phenotype in feet)
***P4.1***	seq[GR38] der(7)t(7;10)(q21.3;q22.3)	N/A	Translocation separates enhancers hs1626, hs1831, eDlx#26, eDlx#24, eExon15, eExon17, eDlx#23, and hs1642 from *DLX5* and *DLX6*											Ectrodactyly of hands and feet
***P4.2***	der(10)t(7;10)(q21.3;q22.3)													Ectrodactyly of hands and feet
***P4.3***	NC_000007.14:g.96261277_qterdelins [NC_000010.11:g.79648260_qter]													Mild brachydactyly of feet
	NC_000010.11:g.79648260_qterdelins [NC_000007.14:g.96261284_qter]													
***P5***	seq[GRCh38] der(7)t(7;12)(q21.3;q23.1)ins(12;12)(q23.1;21.2)^@^	N/A	Translocation separates enhancers hs1626, hs1831, eDlx#26, eDlx#24, eExon15, eExon17, eDlx#23, hs1642, eDlx#19, and eDlx#18 from *DLX5* and *DLX6*											Severe EEC syndrome: bilateral ectrodactyly of hands and feet, facial dysmorphism (i.e., micrognathia, smooth philtrum, thin upper lip, almond-shaped eyes, prominent forehead with V-shaped hairline, and low-set posteriorly rotated and protruding ears), strabismus, cryptorchidism, defect in the atrial septum, global developmental delay, absent speech, and hearing loss
	seq[GRCh38] der(12)inv(12)(q21.2q23.1)t(7;12)(q21.3;q21.2)^%^													

N/A, not applicable; @ - the complex aberration description at the DNA level: NC_000007.14:g.96511147_qterdelins[TGTTAAGAACGTAGTATTACTACCTGTAAAGAACACCTCTCTGAC; NC_000012.11:g.[97709855_98132696;78983925_79478223;98132732_qter]]; % - the complex aberration description at the DNA level: NC_000012.11:g.78983902_79479752delins[GGTAGT;79479753_97703036inv;97704275_qterdelins[GCCTAC;NC_000007.14:g.96513535_qter]]; * - families F6 and F7 were previously published as “Family 1” and “Family 3”, respectively (Tayebi et al., 2014); ** - the previously published *DLX5/6* enhancers (Zerucha et al., 2000; Pennacchio et al., 2006; Verzi et al., 2007; Kouwenhoven et al., 2010; Birnbaum et al., 2012); hs1626 - branchial arches and facial mesenchyme enhancers; hs1831 - heart enhancer; eDlx#26 - forebrain, neural tube, and heart enhancer; eDlx#24 - enhancer in the forebrain, pectoral fin, and heart (zebrafish); eExon15 - limb enhancer and genital tubercle enhancer; eExon17 - limb enhancer; eDlx#23 - enhancer in the forebrain, limb, branchial arches, and ear (otic vesicle); hs1642 - enhancer in the neural tube, hindbrain (rhombencephalon), and forebrain; eDlx#19 - enhancer in the branchial arches; eDlx#18 - enhancer in the branchial arches; eDlx#14 - enhancer in the forebrain, olfactory bulb, and trunk (zebrafish); eDlx#16 - enhancer in the forebrain, olfactory bulb, and caudal fin (zebrafish); BS1 - enhancer in the forebrain, ear, pectoral fin (zebrafish), and limb (mouse); eDlx#8 - enhancer in the somitic muscle (zebrafish); 5K-Del - enhancer in the inner ear (mouse); eDlx#4 - enhancer in the branchial arches (mouse); I56i - enhancer in the forebrain and branchial arches (mouse); I56ii - enhancer in the forebrain.

### 3.2 Karyotyping and microarray-based approaches

#### 3.2.1 Karyotyping

**FIGURE 2:**
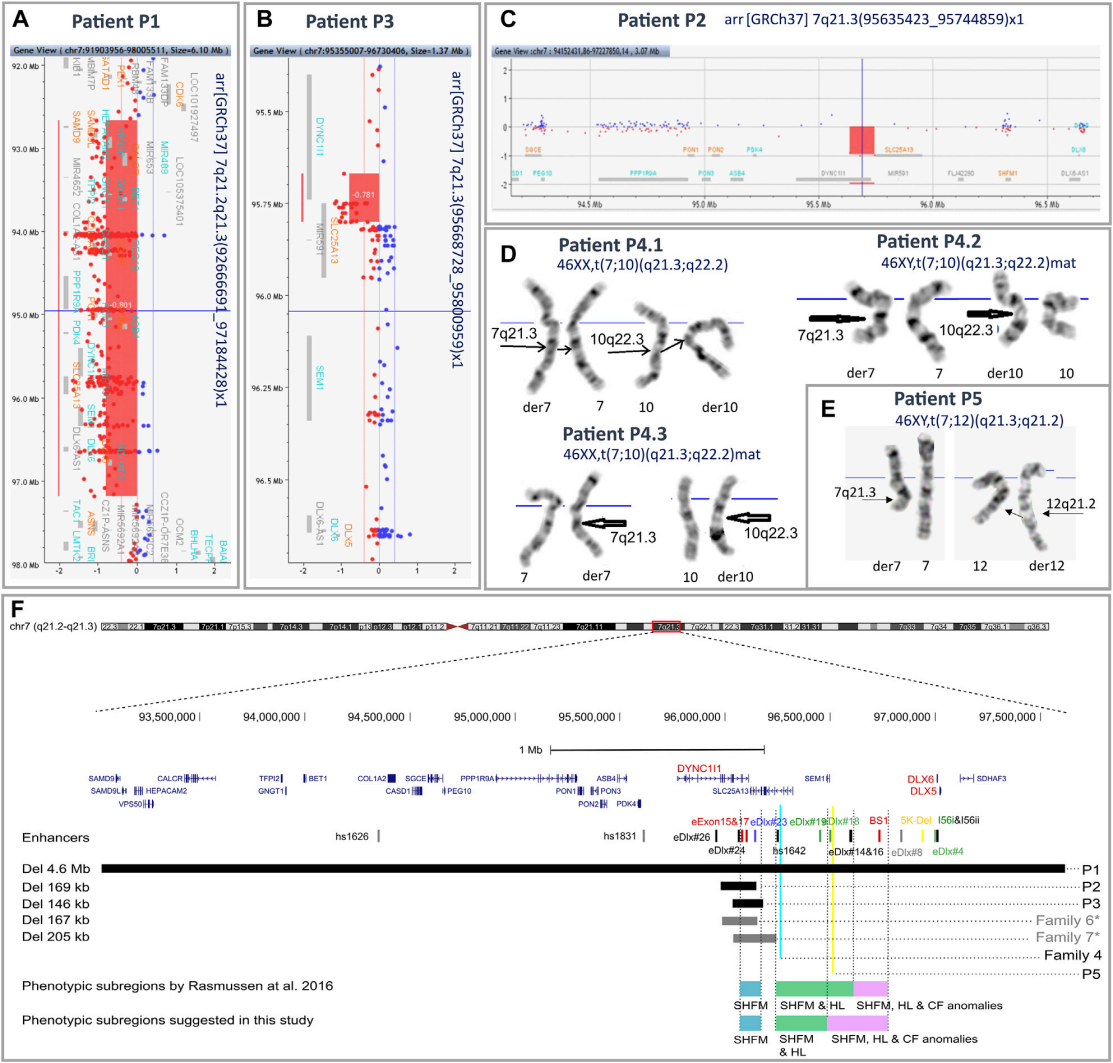
Results of aCGH and karyotyping with an overview of the 7q21.3 genomic region. An overview of aCGH results in the index patients from families 1 **(A)**, 3 **(B)**, and 2 **(C)**. Reciprocal translocations identified in the affected patients from family 4 **(D)** and the index patient from family 5 **(E)** by means of conventional GTG banding at 550 band resolution per haploid genome. **(F)** Mapping of the 7q21.3 rearrangements identified in the described cohort. The previously published *DLX5/6* enhancers are indicated by vertical lines. The colors represent predicted function: red—limb, black—brain, green—branchial arch, blue—otic vesicle, yellow—inner ear, and gray—heart (Zerucha et al., 2000; Pennacchio et al., 2006; Verzi et al., 2007; Kouwenhoven et al., 2010; Birnbaum et al., 2012). Horizontal black and gray bars indicate deleted regions in patients P1–P3 and previously described patients from families 5 and 7 from the Polish cohort, respectively. Blue and yellow vertical lines indicate breakpoints of the translocations identified in affected patients from family 4 and patient P5. Blue, green, and purple horizontal bars represent the phenotypic subregions for SHFM1 associated with isolated SHFM (blue), SHFM and HL (green), and SHFM, HL, and CF anomalies (purple), as proposed by Rasmussen et al., 2016. *—families F6 and F7 were previously published as “Family 1” and “Family 3”, respectively (Tayebi et al., 2014). The genomic coordinates of chromosomal aberrations and enhancers were uploaded on the UCSC Human Genome Browser (GRCh38/hg38)^7^ as custom tracks (Zerucha et al., 2000; Pennacchio et al., 2006; Verzi et al., 2007; Kouwenhoven et al., 2010; Birnbaum et al., 2012; Rasmussen et al., 2016).

**FIGURE 3:**
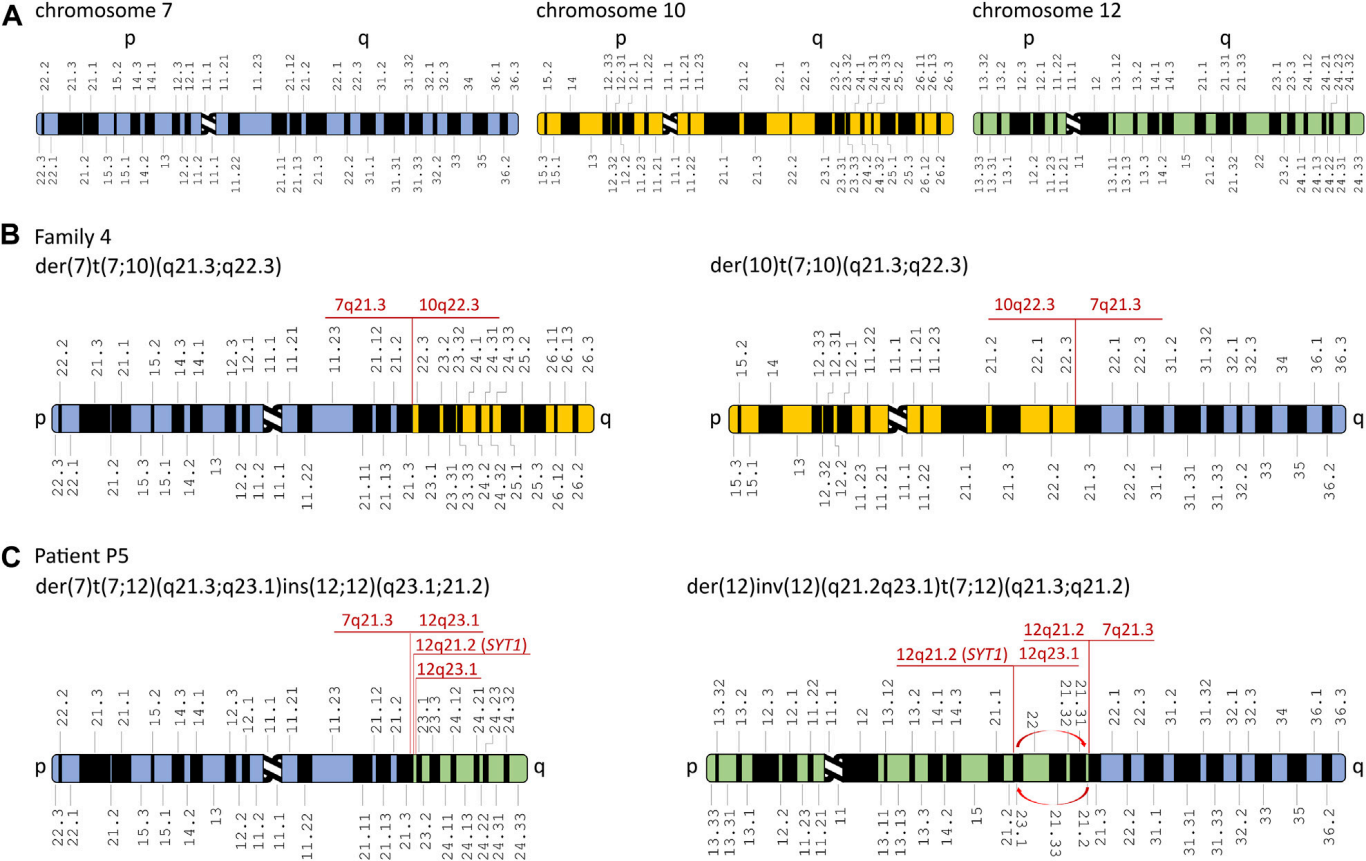
Schematic representation of chromosomal rearrangements identified in the affected patients from families 4 and 5. **(A)** Schematic representation of chromosomes 7, 10, and 12. Derivative chromosomes in the affected patients from family 4 **(B)** and patient P5 **(C)**. Red arrows represent inversion in the derivative chromosome 12. The aberrations’ breakpoints are shown in red. Modified from Idiogram Album: Human copyright ^©^ 1994 David Adler.

Karyotyping revealed balanced translocations: t(7;10)(q21.3;q22.2) in all affected patients from family 4 (patients P4.1, P4.2, and P4.3) (Figure 2D) and t(7;12)(q21.3;q21.2) in the sporadic patient P5 (Figure 2E). A schematic representation of the identified chromosomal aberrations is shown in Figure 3.

#### 3.2.2 Array comparative genomic hybridization and real-time quantitative PCR

Using the array CGH approach, we identified deletions of various sizes at 7q21.3 or 7q21.2–q21.3 in the probands from families 1–3. In the index patient from family 1 (P1), we found a 4.5 Mb deletion at the locus 7q21.2–21.3 (arr[GRCh37] 7q21.2–q21.3(92666691_97184428)), encompassing 25 protein-coding genes, including *DLX5/6*, together with their entire regulatory units (Figure 2A). In the proband P2, we identified a 109 kb deletion at 7q21.3 (arr[GRCh37] 7q21.3(95635423_95744859)x1) (Figure 2C). The proband P3 harbored a 132 kb deletion at the locus 7q21.3 (arr[GRCh37] 7q21.3(95668728_95800959)x1) (Figure 2B). In both cases of P2 and P3, the deletion included fragments of *DYNC1I1* and *SLC25A13* genes and four tissue-specific enhancers. A series of qPCR assays confirmed the 7q21.3 deletions in the probands and excluded their presence in their unaffected parents. Subsequent rounds of qPCR narrowed down the region of the deletions and allowed for the design of primers for breakpoint sequencing (Supplementary Figures S1A–C).

### 3.3 Whole-genome sequencing

The WGS approach enabled us to establish the exact breakpoints of the translocations in the examined patients from families 4 and 5. The mean depth of coverage for patients P4.1, P4.3, and P5 was ×35.9, ×31.2, and ×34.7, respectively; 90.5%, 89.2%, and 88.1% of the genomes of patients P4.1, P4.3, and P5, respectively, were covered by at least 20 uniquely mapping reads. The ISCN description of the aberrations is found in the following section (3.4 Breakpoint Sanger sequencing) and in Table 1 and Supplementary Figure S1E-I. The WGS analysis performed in patient P5 revealed multiple breakpoints contributing to a complex chromosomal aberration involving reciprocal translocation with additional rearrangements, i.e., inversion and reorganization between fragments of the long arm of chromosome 12. The aberration’s breakpoints have been confirmed by PCR followed by Sanger sequencing (Supplementary Figure S1E-I and Table 1). A schematic representation of the identified chromosomal aberrations is shown in Figure 3. The comparative analysis performed between patients P4.1 and P4.3 did not reveal any genetic modifiers that potentially contributed to distinct phenotypic manifestation of the disorder.

### 3.4 Breakpoint Sanger sequencing

Breakpoint sequencing performed in affected patients from all analyzed families enabled us to map the exact size and location of the 7q21.3/7q21.2–q21.3 deletions identified by means of aCGH and structural variants detected using the WGS approach. Description of the variants was prepared according to the ISCN recommendations. The sequencing results are shown in Supplementary Figure S1 and summarized in Table 1.

### 3.5 *SYT1* relative expression in PBMCs of patient P5

Expression analysis of PBMCs of proband P5 revealed a 70% decrease (to 0.3) in the gene expression compared to the mean value of the control samples. The results were statistically significant (*p* < 0.00001) (Supplementary Figure S1D).

## 4 Discussion

In this paper, we present seven newly reported patients from five unrelated Polish families affected by SHFM1 and discuss two previously described families from our SHFM1 cohort (Tayebi et al., 2014). In all presented cases, the disorder resulted from chromosomal structural variants involving deletions or translocations with breakpoints located at the 7q21.2–q21.3 locus. In eight patients, the disease-causing mechanism was associated with a regulatory position effect consisting of the deletion or repositioning of tissue-specific enhancers. In one patient, the condition resulted from a large deletion encompassing both the regulatory elements and the target genes *DLX5*/*6*. The genotype–phenotype correlation presented herein is consistent with the previously published phenotypic subregions within the SHFM1 locus (Rasmussen et al., 2016) for all analyzed cases except one (patient P5). Rasmussen et al. (2016) presented a comprehensive review of the literature focusing on the genotype–phenotype correlation of 100 patients from 32 SHFM1 families, in which the disorder was caused by SVs at the 7q21.3 locus. The studied patients were grouped according to the clinical manifestation of the disorder, i.e., isolated SHFM, SHFM with HL, and SHFM with HL and CF anomalies. A thorough analysis of the variant’s breakpoints performed for the distinct phenotypic groups of patients, in relation to the location of the *DLX5/6* tissue-specific enhancers, allowed for mapping each group to a common genomic region, thus suggesting the occurrence of three phenotypic subregions within the SHFM1 locus (Rasmussen et al., 2016). The region located most distant from *DLX5/6* overlaps with the critical SHFM1 locus, encompassing eExon15 and eExon17 limb enhancers along with the eDlx#23 multi-tissue enhancer, and is associated with isolated SHFM. The intermediate subregion is related to SHFM with HL with no CF anomalies and contains five forebrain and/or branchial arch enhancers (hs1642, eDlx#19, eDlx#18, eDlx#16, and eDlx#14). The third subregion, associated with the full spectrum of clinical manifestation, i.e., SHFM with HL and CF anomalies, localizes closest to *DLX5/6* and encompasses a single BS1 enhancer that displays activity in the limb, ear, and forebrain (Rasmussen et al., 2016) (Figure 2F). It has been shown that BS1 interacts with the *Dlx6* promoter and with two *Dlx5/6* enhancers, namely, I56i and I56ii, which regulate the gene expression in the forebrain and branchial arches (Zerucha et al., 2000; Ghanem et al., 2003; Kouwenhoven et al., 2010). According to the authors, the disruption of these interactions explains the hearing loss and craniofacial anomalies diagnosed in the third phenotypic group of SHFM1 patients (reviewed in Rasmussen et al. (2016)). The analysis of the results obtained for our patients allows us to reevaluate the position of the centromeric boundary of the third subregion that, accordingly, should also include the branchial arch enhancer eDlx#18 (Figure 2F). Our suggestion is substantiated by the results of the breakpoint sequencing analysis of the reciprocal t(7;12)(q21.3;q21.2) translocation found in the most severely affected patient in our cohort - P5. The patient displayed EEC syndrome with multiple additional abnormalities unspecific for SHFM1. The results of WGS, followed by Sanger sequencing evaluation, showed a complex chromosomal aberration involving not only a reciprocal translocation between long arms of chromosomes 7 and 12 but also additional reorganization and inversion of segments originating from chromosome 12 (see Figure 3C, Table 1, and Supplementary Figure S1G-I for the variant description). The translocation’s breakpoint on chromosome 7 was located within the SHFM1 7q21.3 locus and resulted in the dislocation of a number of tissue-specific enhancers, i.e., the limb, branchial arch (eDLX#19 and eDlx#18), brain, heart, and ear (otic vesicle) from the *DLX5/6* genes (Figure 2F). Nevertheless, the interaction between BS1 and the *DLX5/6* locus remained intact, yet the patient manifested CF anomalies, i.e., micrognathia and low-set posteriorly rotated and protruding ears (Figures 1O,P). Accordingly, we concluded that BS1 activity may not be sufficient to maintain proper *DLX5/6* expression in the branchial arches and that possibly at least one other distant branchial arch-specific enhancer, i.e., eDLx#18 or 19, would probably need to be active in order to prevent the manifestation of CF anomalies. While the EEC syndrome observed in patient P5 resulted clearly from the 7q21.3 translocation breakpoint, the additional phenotypic features, which were not typical of SHFM1 clinical manifestation, resulted from the breakpoint within chromosome 12 in the second intron of the *SYT1* gene (OMIM 185605; NM_005639.3). *SYT1* encodes synaptotagmin 1, a protein that plays a critical role in mediating synchronous calcium-dependent neurotransmitter release via triggering the synaptic vesicle fusion. Heterozygous missense loss-of-function or dominant-negative mutations in *SYT1* result in Baker-Gordon syndrome (BAGOS) (OMIM 618218), a neurodevelopmental disorder characterized by a global developmental delay, poor or absent speech, infantile hypotonia, ophthalmic abnormalities (i.e., epicanthal folds, almond-shaped eyes, strabismus, and nystagmus), facial dysmorphic features (e.g., prominent high forehead with V-shaped hairline, smooth philtrum, and thin upper lip), hyperkinetic movements, gastrointestinal abnormalities, sleep apnea, and EEG abnormalities in the absence of overt seizures (Baker et al., 2015; 2018). Interestingly, patient P5 exhibited symptoms that were overlapping with the BAGOS phenotypic manifestation, i.e., global developmental delay, absent speech, hypotonia, defect in the atrial septum, high forehead with V-shaped hairline, strabismus, almond-shaped eyes, smooth philtrum, and thin upper lip (Figure 1O-Q), that most likely resulted from *SYT1* loss-of-function. Up to date, only five variants have been reported to cause BAGOS, all of which are missense mutations clustered in one of the two highly conserved calcium-binding domains of the synaptotagmin-1 protein, the C2B domain (Baker et al., 2015; 2018; Cafiero et al., 2015). These pathogenic variants were associated with variable phenotypic severity of the disorder relating mostly to the spectrum of neurological abnormalities. This mutation-specific impact on protein expression, function, and localization at nerve terminals resulted in a variable degree of disturbance to synaptic vesicle kinetics (Baker et al., 2018). At least one of the reported mutations (I368T) has been shown to act through a dominant negative mechanism (Baker et al., 2015); however, the mechanism of action of the other variants remains elusive and requires further studies. Nevertheless, for mutations associated with the mildest neurodevelopmental impairment (e.g., M303K), a loss-of-function mechanism is considered. The results of our findings support this hypothesis since the translocation breakpoint identified in the patient P5 was mapped to the second intron of *SYT1,* which is a part of gene’s 5′-UTR. This, in turn, leads to the dislocation of the entire coding part of *SYT1* from its promoter. This regulatory mutation resulted in a 70% reduction in gene expression in the proband’s PBMCs compared to controls (Supplementary Figure S1D), pointing to its loss-of-function effect. Apart from the typical BAGOS symptoms, our patient was diagnosed with the atrial septal defect that, surprisingly, was also observed in a single patient from the cohort described by Baker et al., 2018 (patient 4). Our findings, therefore, confirm that this defect may be a characteristic feature of BAGOS.

Although developmental delay (DD) was reported in 33% of SHFM1 patients (Elliott and Evans, 2006), the abnormality was restricted to large deletions or translocations of which at least one breakpoint was mapped outside the SHFM1 locus (Haberlandt et al., 2001; Bernardini et al., 2008; Saitsu et al., 2009; van Silfhout et al., 2009; Brown et al., 2010; Vera-Carbonell et al., 2012; Delgado and Velinov, 2015; Sivasankaran et al., 2016; Rai et al., 2019). Since the feature was absent in patients carrying a deletion limited to the *DLX5/6* TAD, neither was it identified in patients harboring SNVs in *DLX5* (reviewed in Rasmussen et al. 2016)), it seems unlikely that *DLX5/6* contribute to DD. Consequently, the global DD observed in patient P5 was most likely a manifestation of BAGOS rather than SHFM1. In addition to this finding, DD was also not present in the sporadic patient P1 from our cohort, who carried a large 4.6 Mb deletion encompassing the entire SHFM1 locus, including the *DLX5/6* genes and their regulatory units. As suspected, the patient was diagnosed with the full spectrum of SHFM1 symptoms, including the EEC syndrome (i.e., bilateral ectrodactyly of hands and feet, enamel and nail hypoplasia, and tendency for tooth decay), hearing loss, and craniofacial anomalies with the characteristics of the 7q21.3 locus, namely, micrognathia and malformation of the ears (i.e., overfolded helix) (Figures 1A–D). Among the deleted genes, 10 were disease-associated; however, only one, namely, *SGCE* (OMIM: 604149), was likely dosage-sensitive and associated with an autosomal dominant phenotype. Heterozygous truncating mutations in the *SGCE* gene lead to myoclonus-dystonia syndrome (OMIM: 159900), characterized by myoclonic jerks affecting mainly proximal muscles. Nevertheless, the gene is maternally imprinted and, consequently, expressed only from the paternal allele; thus, reduced penetrance is observed upon maternal transmission (Schüle et al., 2004). This finding presumably explains the lack of phenotypic manifestation of myoclonus-dystonia syndrome in patient P1.

Unlike the aforementioned patient P5 with EEC, harboring the t(7;12)(q21.3;q21.2), all carriers of the t(7;10)(q21.3;q22.2) translocation from family 4 (P4.1, P4.2, and P4.3) were diagnosed with an isolated SHFM with reduced penetrance. The index patient P4.1—the mother and her son P4.2 manifested bilateral ectrodactyly of hands and feet, whereas the daughter (P4.3) was affected with mild brachydactyly of feet. When comparing the location of the rearrangement breakpoints at the 7q21.3 locus in both families, we found that the disrupted region was diversified by only two branchial arch enhancers, eDlx#19 and eDlx#18, yet associated with significantly distinct phenotypic expression. Both enhancers remained intact within *DLX5/6* TAD in individuals from family 4 and dislocated from the target genes in patient P5. This finding further supports the crucial role of eDlx#19 and/or eDlx#18 in the pathogenesis of craniofacial anomalies and EEC syndrome. Thorough analysis of WGS results, aiming to search for genetic modifiers associated with reduced penetrance in the members of family 4, did not reveal any variants that could potentially explain this phenomenon. Nevertheless, brachydactyly - especially phalangeal aplasia or hypoplasia - is, same as SHFM, a form of limb reduction defect and, in this case, can be regarded as a manifestation of the SHFM spectrum, most likely resulting from 7q21.3 locus disruption. Incomplete penetrance is relatively common in SHFM1 familial cases; however, it is limited only to isolated forms and associated with a disruption of eExon15, eExon17, and eDlx#23 enhancers. There is a clear sex bias among the mutation carriers since it has been demonstrated that the penetrance is higher in males vs. females and that the disorder is overtransmitted from affected fathers to sons (reviewed in Rasmussen et al., (2016)). This observation can possibly result from the functionally supported hypothesis of *DLX5/6* maternal imprinting in osteoblasts (Rattanasopha et al., 2014).

Two unreported sporadic patients from our cohort (P2 and P3), along with individuals from two previously published Polish families (family 6 and 7 - this study; published as family 1 and 3, respectively, in Tayebi et al. (2014)), who harbored the smallest microdeletions, were diagnosed with isolated SHFM. In all cases, the deletion encompassed a common cluster of regulatory elements, i.e., two limb enhancers, eExon15 and eExon17, along with two multiple-tissue enhancers, eDLX#24 and eDLX#23, which was consistent with the “isolated SHFM” phenotypic subdomain proposed by Tayebi et al. (2014) and Rasmussen et al. (2016). Interestingly, despite the highly overlapping location of the CNVs (especially for patient P2 and members of family 6 - see Figure 2F), the cohort presented variable expressivity as ectrodactyly was limited to feet in P2 and P3 individuals (hands were unaffected), whereas all affected members of families 6 and 7 manifested SHFM in all four extremities. Additionally, the disorder was highly variable among individuals from family 7, ranging from a severe cleft to mild hypoplasia of the central digit and mild defect of the feet. Variable phenotypic expression of identical or highly similar genetic mutations is relatively frequent in many limb malformation phenotypes. In a number of cases, this phenomenon could be explained by differences in the gene’s regulatory environment, while in other studies, it remains unexplained and requires further studies involving epigenetic modifications and contributions from environmental factors.

In conclusion, in this paper, we present seven novel cases from five Polish families diagnosed with variable features of the SHFM1 spectrum, all resulting from chromosomal rearrangements at the 7q21.3/7q21.2–q21.3 locus. We performed a thorough genotype–phenotype correlation of the cyto-molecular results described herein against previously suggested subregions of the SHFM1 locus, which enabled us to refine the regulatory units, critical for specific clinical manifestation of the disorder. Consequently, we suggest that the dislocation of the branchial arch-specific enhancers, eDlx#18 and/or eDlx#19 from the *DLX5/6* target genes, is critical for the pathogenesis of craniofacial anomalies in EEC syndrome. Additionally, we present the first evidence that a loss-of-function regulatory mutation within the *SYT1* gene results in the BAGOS-like phenotype since these features, along with EEC syndrome, were present in a sporadic patient harboring a reciprocal translocation that disrupted both SHFM1 and *SYT1* loci. In our work, we highlight the need for sequence-based approaches in defining the exact breakpoints of pathogenic SVs in the molecular diagnostics of patients whose features point to the disruption of certain tissue-specific enhancers, such as in SHFM1.

## Data Availability

The raw data supporting the conclusions of this article will be made available by the authors, without undue reservation.

## Footnotes

1
https://www.primer3plus.com/

2
https://genetools.org/SNPCheck/snpcheck.htm

3
https://www.ensembl.org/Multi/Tools/Blast

4
https://github.com/brentp/smoove

5
https://varsome.com/

6
https://www.ncbi.nlm.nih.gov/tools/primer-blast/index.cgi

7 Human Genome Browser, that is: https://genome-euro.ucsc.edu/index.html
