# Supplementary material for: A genotype–phenotype correlation in split-hand/foot malformation type 1: further refinement of the phenotypic subregions within the 7q21.3 locus
Source: Front Mol Biosci. 2023 Oct 17;10:1250714. doi: 10.3389/fmolb.2023.1250714 (PMC10616856; doi:10.3389/fmolb.2023.1250714)
Supplement: Supplementary file 1 [file DataSheet2.pdf]

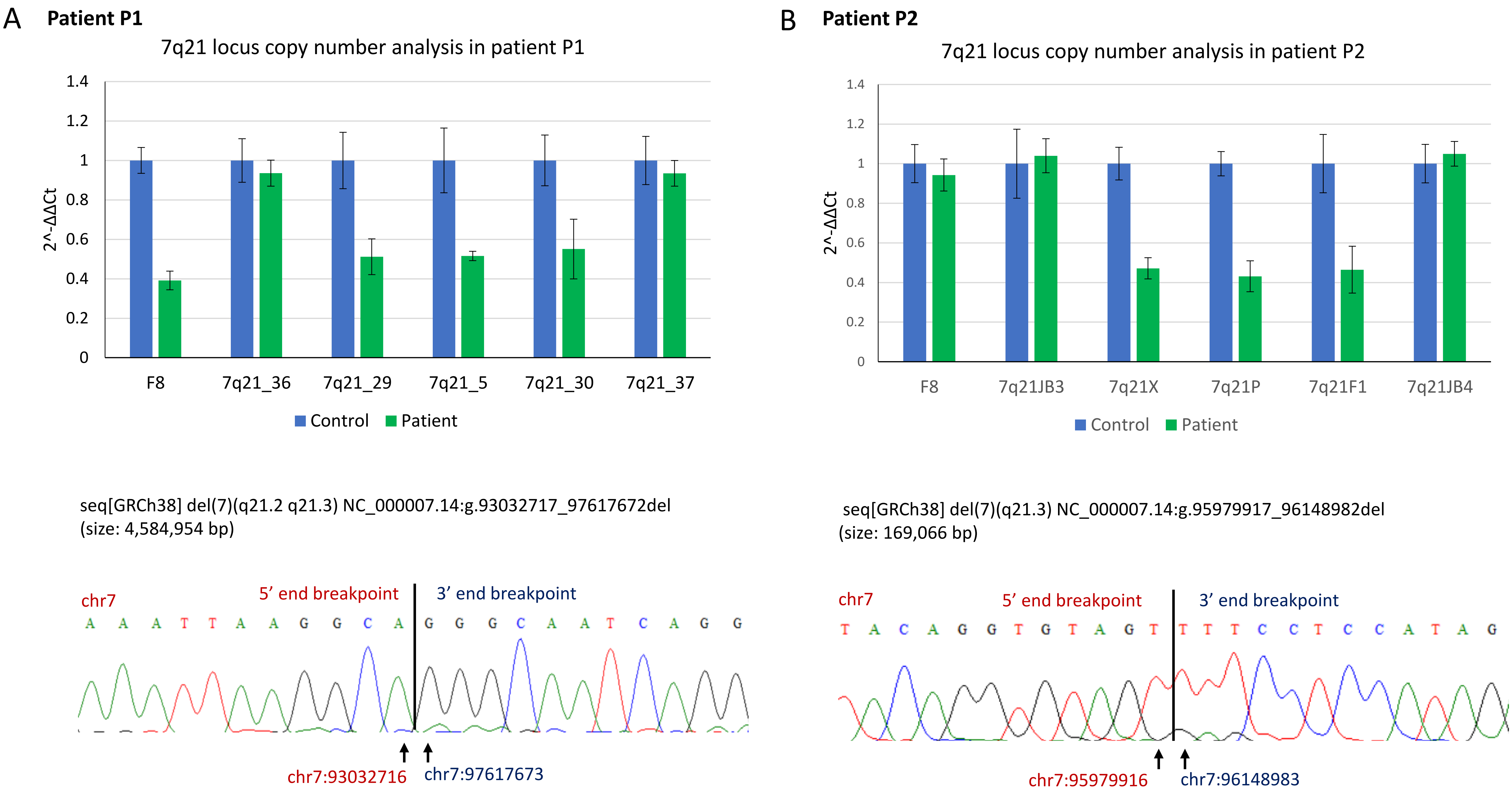
A
Patient P1
7q21 locus copy number analysis in patient P1
2^-ΔΔCt
1.4
1.2
1
0.8
0.6
0.4
0.2
0
F8
7q21_36
7q21_29
7q21_5
7q21_30
7q21_37
Control
Patient
seq[GRCh38] del(7)(q21.2 q21.3) NC_000007.14:g.93032717_97617672del
(size: 4,584,954 bp)
chr7
5’ end breakpoint
3’ end breakpoint
A A A T T A A G G C A G G G C A A T C A G G
chr7:93032716
chr7:97617673
B
Patient P2
7q21 locus copy number analysis in patient P2
2^-ΔΔCt
1.4
1.2
1
0.8
0.6
0.4
0.2
0
F8
7q21JB3
7q21X
7q21P
7q21F1
7q21JB4
Control
Patient
seq[GRCh38] del(7)(q21.3) NC_000007.14:g.95979917_96148982del
(size: 169,066 bp)
chr7
5’ end breakpoint
3’ end breakpoint
T A C A G G T G T A G T T T T C C T C C A T A G
chr7:95979916
chr7:96148983

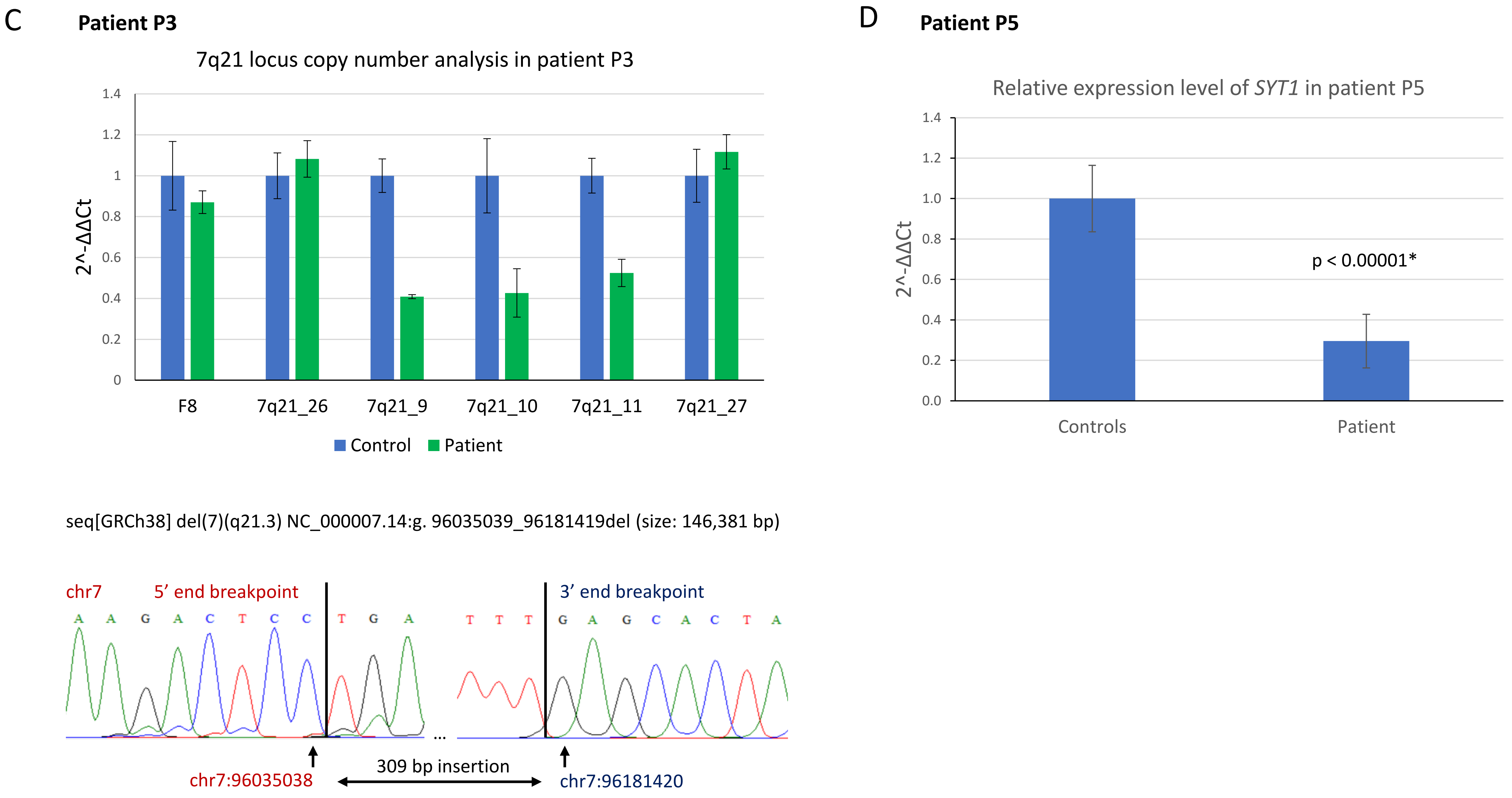
C
Patient P3
7q21 locus copy number analysis in patient P3
2^-ΔΔCt
1.4
1.2
1
0.8
0.6
0.4
0.2
0
F8
7q21_26
7q21_9
7q21_10
7q21_11
7q21_27
Control
Patient
D
Patient P5
Relative expression level of SYT1 in patient P5
2^-ΔΔCt
1.4
1.2
1.0
0.8
0.6
0.4
0.2
0.0
p < 0.00001*
Controls
Patient
seq[GRCh38] del(7)(q21.3) NC_000007.14:g. 96035039_96181419del (size: 146,381 bp)
chr7
5' end breakpoint
A A G A C T C C T G A
3' end breakpoint
T T T G A G C A C T A
...
chr7:96035038
309 bp insertion
chr7:96181420

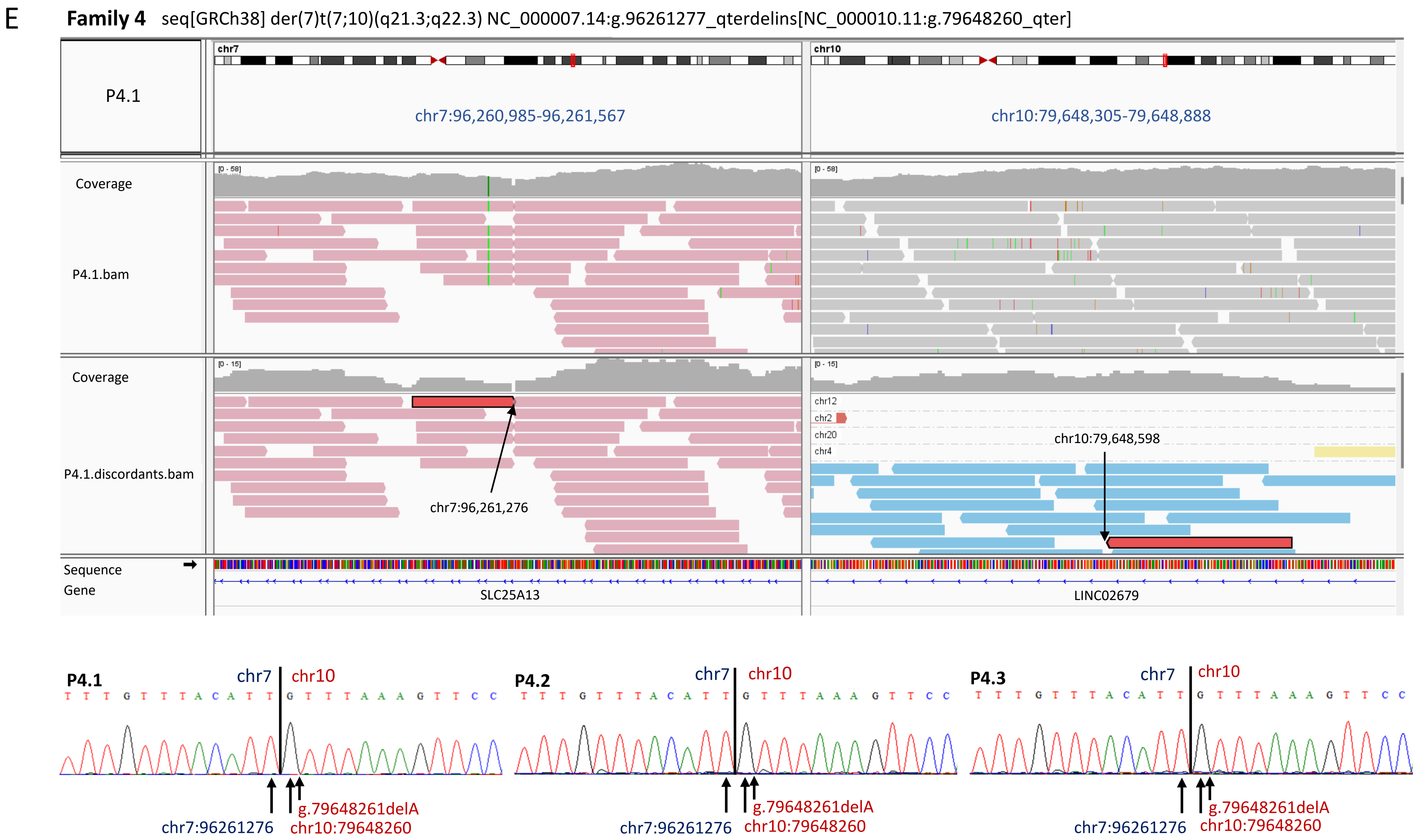
E
Family 4 seq[GRCh38] der(7)t(7;10)(q21.3;q22.3) NC_000007.14:g.96261277_qterdelins[NC_000010.11:g.79648260_qter]
P4.1
chr7
chr7:96,260,985-96,261,567
chr10
chr10:79,648,305-79,648,888
Coverage
P4.1.bam
[0 - 58]
Coverage
P4.1.discordants.bam
[0 - 15]
chr12
chr2
chr20
chr4
chr7:96,261,276
chr10:79,648,598
Sequence
Gene
SLC25A13
LINC02679
P4.1
P4.2
P4.3
chr7
chr10
T T T G T T T A C A T T G T T T A A A G T T C C
chr7:96261276
g.79648261delA
chr10:79648260

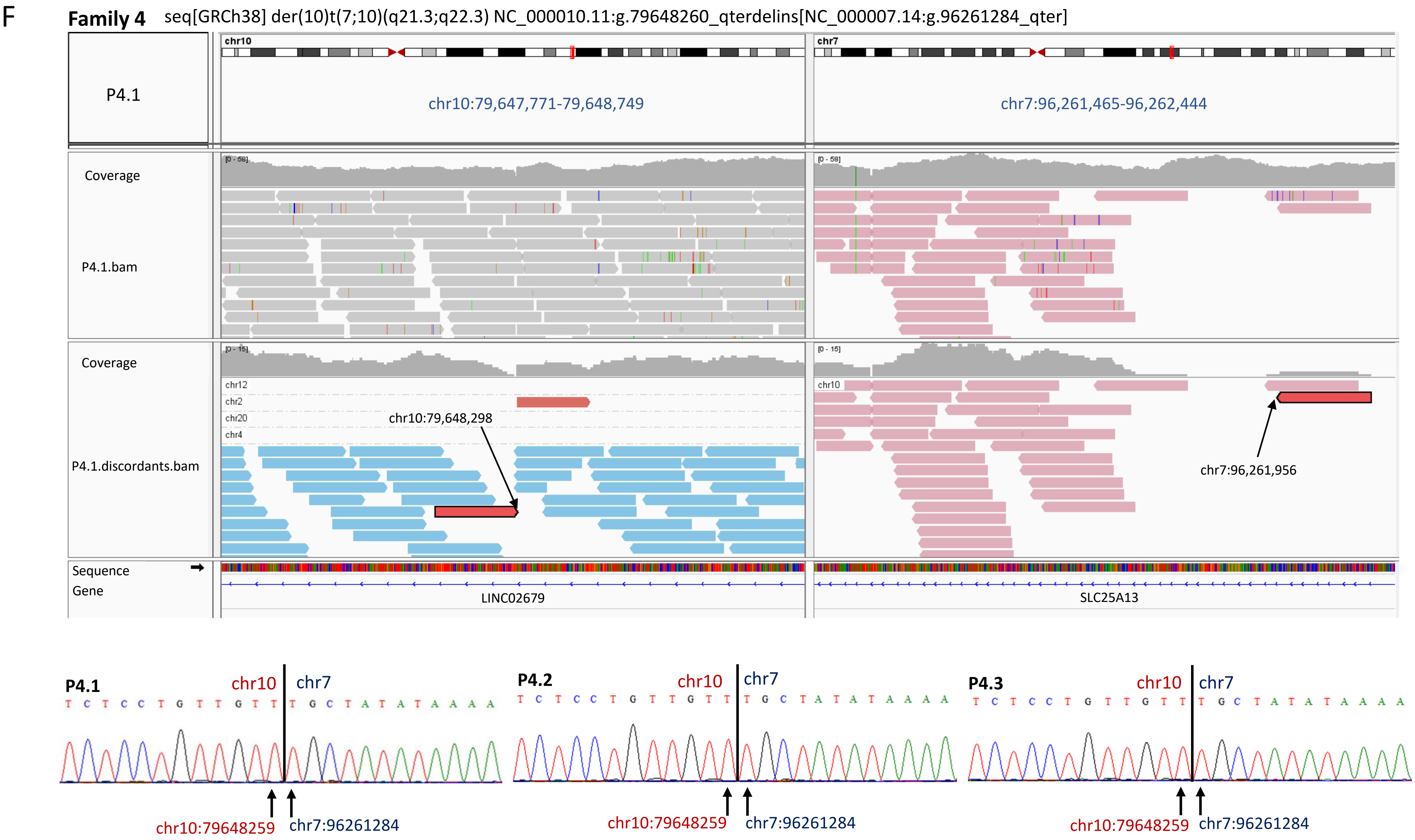
F
Family 4
seq[GRCh38] der(10)t(7;10)(q21.3;q22.3) NC_000010.11:g.79648260_qterdelins[NC_000007.14:g.96261284_qter]
P4.1
chr10
chr7
chr10:79,647,771-79,648,749
chr7:96,261,465-96,262,444
Coverage
P4.1.bam
Coverage
P4.1.discordants.bam
chr12
chr2
chr20
chr4
chr10
chr10:79,648,298
chr7:96,261,956
Sequence
Gene
LINC02679
SLC25A13
P4.1
P4.2
P4.3
chr10
chr7
T C T C C T G T T G T T T G C T A T A T A A A A
chr10:79648259
chr7:96261284

G **Patient P5** seq[GRCh38] der(7)t(7;12)(q21.3;q23.1)ins(12;12)(q23.1;q21.2)

NC_000007.14:g.96511147_qterdelins[TGTTAAGAACGTAGTATTACTACCTGTAAAGAACACCTCTCTGAC;NC_000012.11:g.[97709855_98132696;78983925_79478223;98132732_qter]]

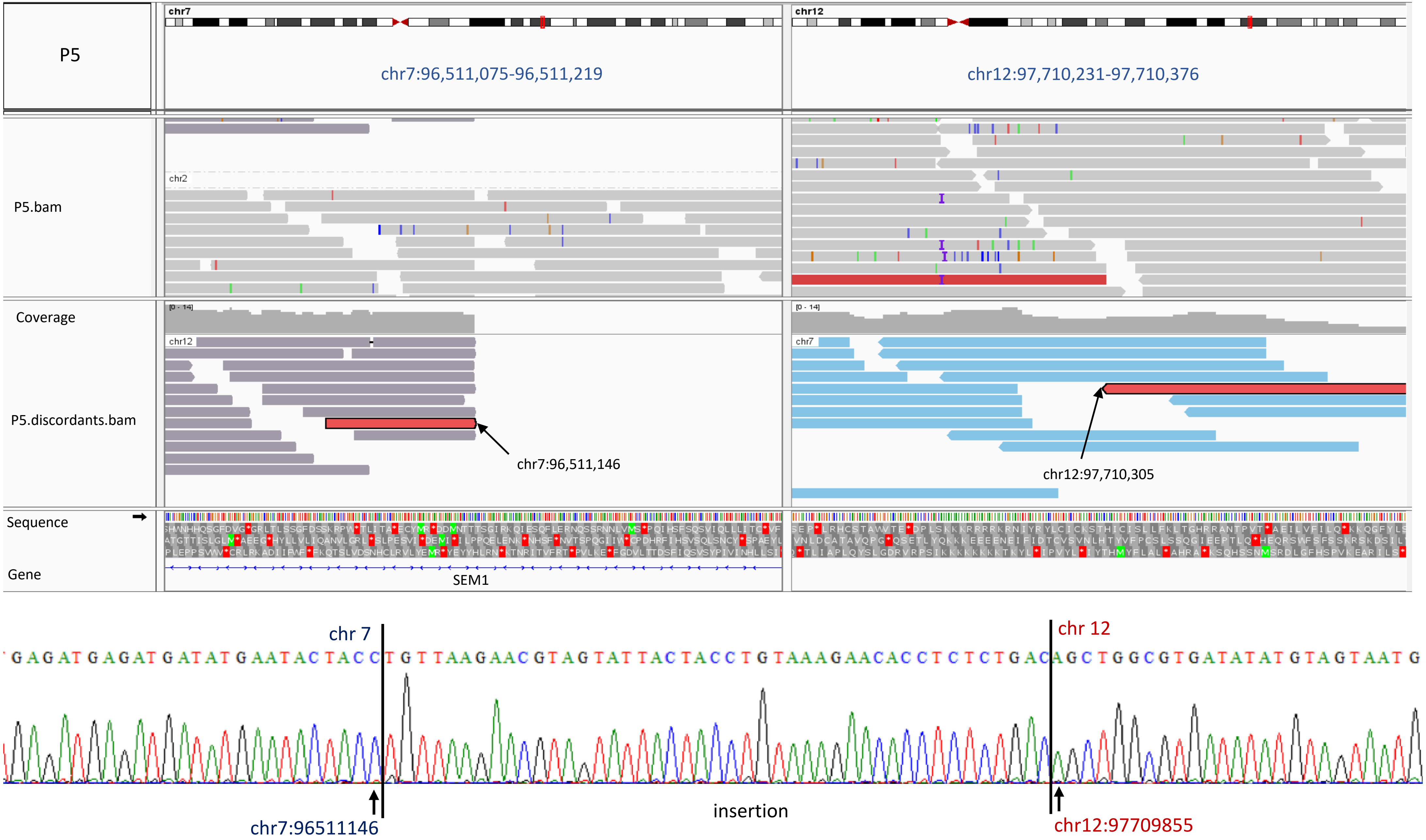

H **Patient P5** seq[GRCh38] der(7)t(7;12)(q21.3;q23.1)ins(12;12)(q23.1;q21.2)

NC_000007.14:g.96511147_qterdelins[TGTTAAGAACGTAGTATTACTACCTGTAAAGAACACCTCTCTGAC;NC_000012.11:g.[97709855_98132696;78983925_79478223;98132732_qter]]

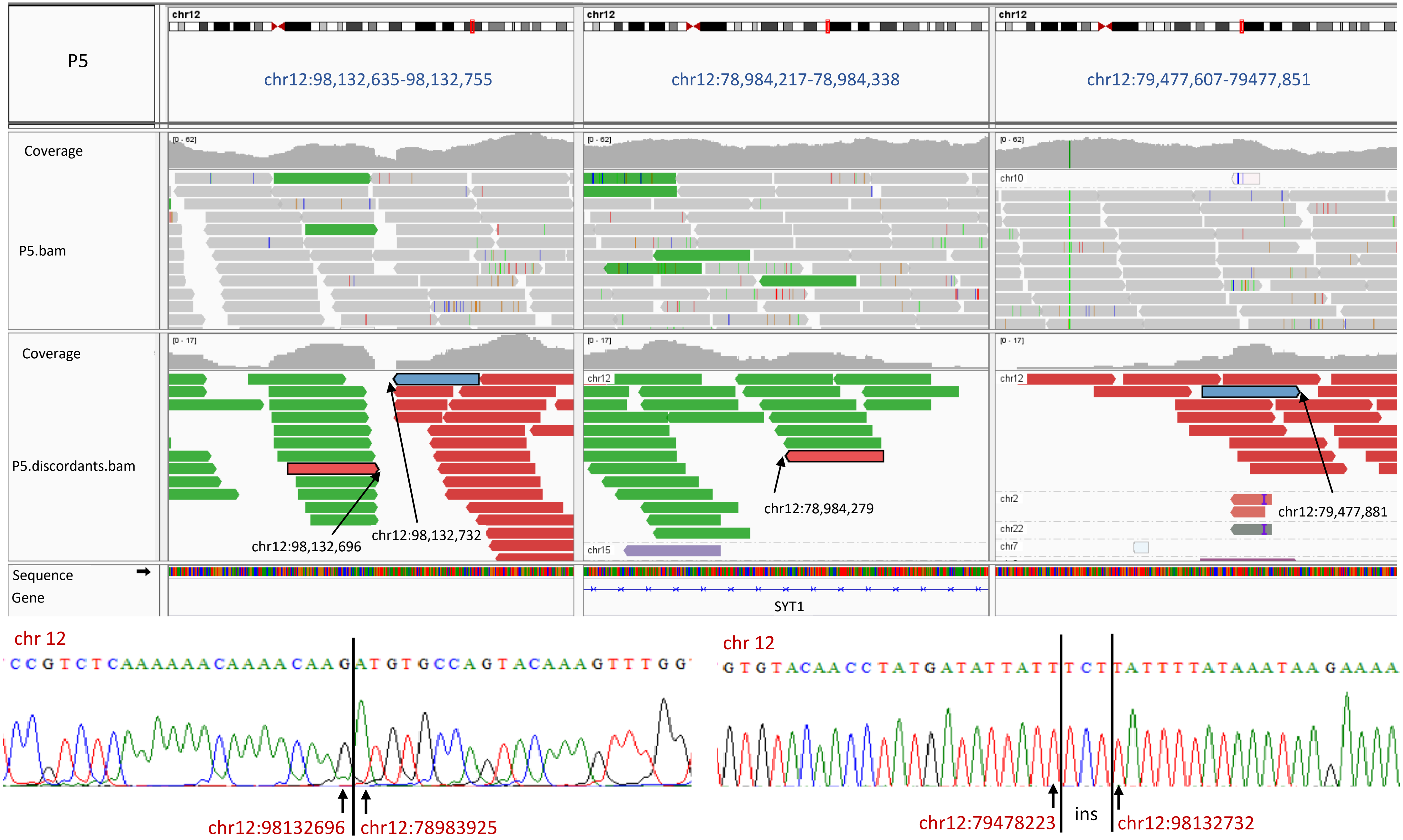

Patient P5 seq[GRCh38] der(12)inv(12)(q21.2q23.1)t(7;12)(q21.3;q21.2)

NC_000012.11:g.78983902_79479752delins[GGTAGT;79478480_97703036inv;97704275_qterdelins[GCCTAC;NC_000007.14:g.96513535_qter]]

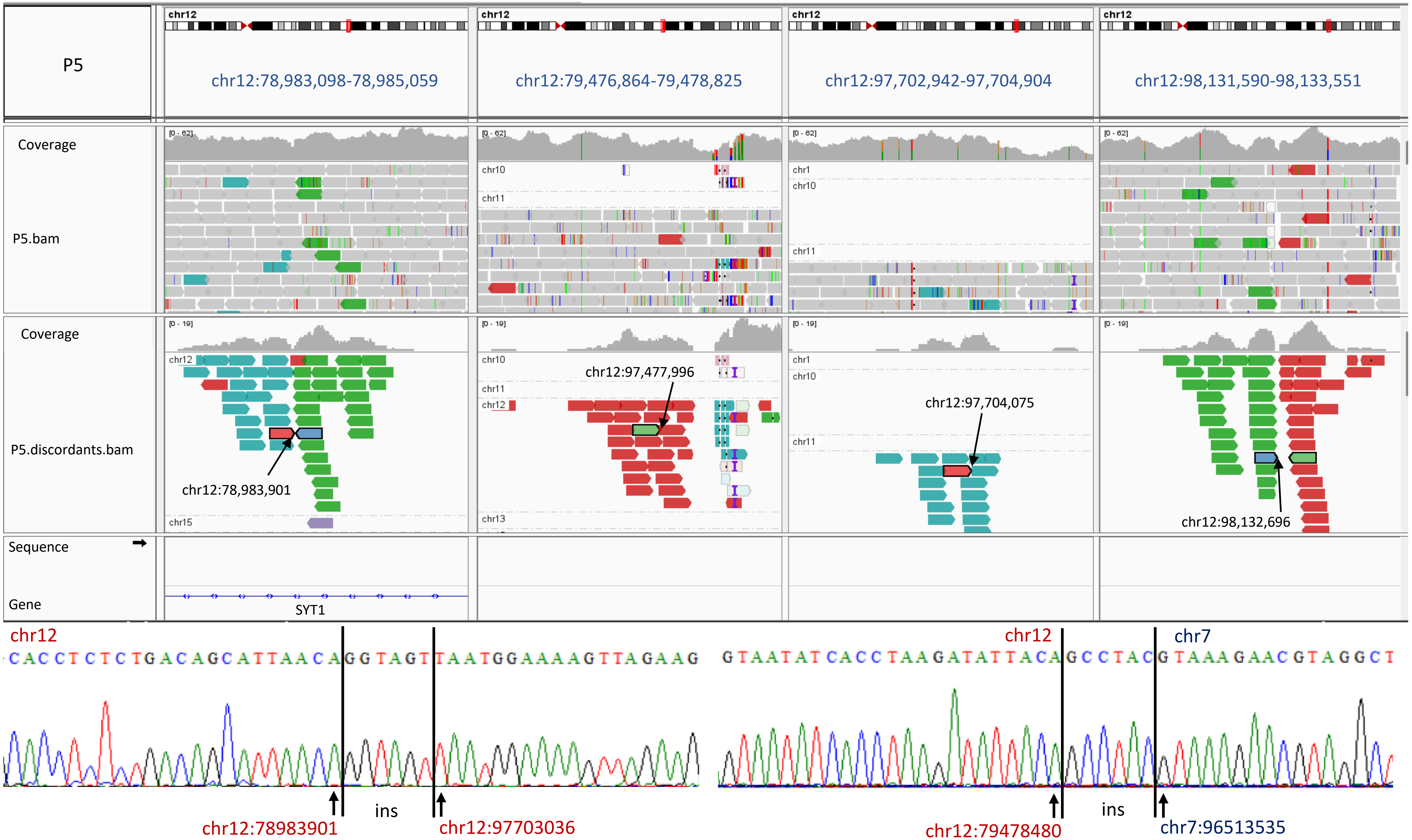

Supplementary Figure 1. Presentation of qPCR, WGS, and Sanger sequencing results in the examined cohort. **(A-C)** qPCR results showing one copy within the analyzed region of the heterozygous deletion (~ 0.5 fold decrease) in patients P1 **(A)**, P2 **(B)** and P3 **(C)**. Patients and controls are depicted in green and blue respectively. Error bars represent standard deviation. Bottom panels of sections **A-C** show results of Sanger sequencing of the 7q21.3 deletions' breakpoints. Black arrows indicate the nucleotide coordinates. Note an insertion of 304 bp fragment at the site of the breakpoints in patient P3 **(C)**. **(D)** Relative expression level of *SYT1* in PBMCs of patient P5 and controls indicate a 70% decrease (to 0.3) of the gene expression in the patient compared to the mean value of control samples. Error bars represent standard deviation. *Statistical significance was calculated using one-tailed single sample z-test. The result is significant at $p < 0.01$. **(E, F – upper panel)** WGS results showing the breakpoints of reciprocal translocation identified in the index patient from family 4 (P4.1) on derivative chromosome 7 **(E)** and derivative chromosome 10 **(F)**. **(E, F – lower panel)** Validation of WGS results by Sanger sequencing performed in patients P4.1, P4.2 and P4.3. **(G-I – upper panel)** WGS results showing the breakpoints of rearrangements on the derivative chromosome 7 **(G, H)** and derivative chromosome 12 **(I)** in patient P5 and the corresponding results of Sanger sequencing validation **(lower panels)**. Note a 45 bp **(G)**, 3 bp **(H)** and two 6 bp **(I)** insertions (ins) within the rearranged sequence. Black arrows indicate the nucleotide coordinates.
