# Supplementary material for: A genotype–phenotype correlation in split-hand/foot malformation type 1: further refinement of the phenotypic subregions within the 7q21.3 locus
Source: Front Mol Biosci. 2023 Oct 17;10:1250714. doi: 10.3389/fmolb.2023.1250714 (PMC10616856; doi:10.3389/fmolb.2023.1250714)
Supplement: Supplementary file 2 [file DataSheet1.PDF]

Table S1. qPCR oligonucleotide primers used to validate and narrow down CNVs

Primer	Sequence (5’ to 3’)	Genomic coordinates (Hg38)	Target
Reference genes			
ALB_conF	TGAAATGGCTGACTGCTGTG	chr4:73,408,650-73,408,732	Autosomal reference *ALB*
ALB_conR	GGAGGTTTGGGTTGTCATCT		
F8_conF	TTTCCATTCAACACCTCAGTCGT	chrX:154,999,491-154,999,575	X chromosome reference *F8*
F8_conR	GCCTTGGCTTAGCGATGTTG		
Index patient P1			
7q21_F1	ATCTCCATGGAAGGCAAGTG	chr7:93,010,744-93,010,830	Normal 5’ region
7q21_R1	ATCAGGAAACCATGGGTGAG		
7q21_F2	TCTGAGAGGTGCTTACCCTGA	chr7:93,021,639-93,021,718	
7q21_R2	TCTGCTGGTTTGACCTTTCC		
7q21_F3	ACTGCTCCTGGCATGAAACT	chr7:93,029,914-93,029,999	
7q21_R3	CTCTGTGCATTCATGCTGGT		
7q21_F12	CCTAGCTTGAGAGGCCAACA	chr7:93,031,142-93,031,225	
7q21_R12	GTCTTGGGGATGCTTCTTGT		
7q21_F35	GCACAGCATGGTAAACTGGA	chr7:93,031,981-93,032,081	
7q21_R35	CCTTGATTTTGAGCCTATGGA		
7q21_F36	GGACCTGAACTCAACACTGGA	chr7:93,032,409-93,032,506	
7q21_R36	ACGTGGCAAGGAGAAGAATG		
7q21_F29	AGGCTCAGGACCAGGTGAAT	chr7:93,033,299-93,033,402	Deleted region
7q21_R29	GGAGTTTCTCCTCAGTTTTCTGG		
7q21_F15	GTGACAGAGGGGCTAAAGCA	chr7:93,036,236-93,036,316	
7q21_R15	GCTTTCCCCAGGAAGAGTTC		
7q21_F4	CTGTTTCAGTGAATGCCACAT	chr7:97,572,725-97,572,808	
7q21_R4	TCCCCAGGGAGCTCAATTA		
7q21_F5	CAGCACCATGAAGGAGATGA	chr7:97,596,062-97,596,142	
7q21_R5	GCCTCAGAGAAGGTCTTCCA		
7q21_F17	TCTAGGCAAGGTGAGCAAGG	chr7:97,600,905-	

7q21_R17	AGAGGAACGCAGCCAAATTA	97,600,990	
7q21_F19	CCTGGCAATTGGGTTTAACA	chr7:97,608,353-97,608,449	
7q21_R19	TGCTCCAAGGTGCAAAACTA		
7q21_F20	TTCCCCTTGCACCTAAACAG	chr7:97,612,991-97,613,078	
7q21_R20	AATCCAGGGCAAGGCTTTAT		
7q21_F30	TTTGTTGGGGAGAGGACATC	chr7:97,613,535-97,613,620	
7q21_R30	TGTGCTTGCCGTGTTTAGTG		
7q21_F37	GGAGTGCTTACAGTGGGGAGT	chr7:97,620,018-97,620,104	Normal 3' region
7q21_R37	CCAGCCATTTTTCATCATGT		
7q21_F32	GCCACCAAGAAGCAAAGGTA	chr7:97,620,762-97,620,851	
7q21_R32	AATTGGTCTGTGTGGCTGCT		
7q21_F33	GACACATGGCCAAGAACTCA	chr7:97,623,426-97,623,514	
7q21_R33	TTTTCCCCTCACGCTACCTA		
7q21_F34	TGATGGCAGCTTGAGAGAGA	chr7:97,624,325-97,624,413	
7q21_R34	TTCTTTGGGCTCACAAGGTT		
7q21_F6	TTTCTGCCAATCCATTCTCC	chr7:97,625,948-97,626,036	
7q21_R6	CACTGAGTGGCTCAAGGTGA		
Index patient P2			
7q21A_F	CCTTTCCAAGCATCTGGTGT	chr7:95,808,061-95,808,148	Normal 5' region
7q21A_R	GTGACAGTGGCCAGTCGATA		
7q21S_F	GTCTCAGCAACCACAGCAAA	chr7:95,933,280-95,933,367	
7q21S_R	TGAACAAACAGACTGGCATTG		
7q21T_F	GGCCTTAGCCTAGCTTCCAT	chr7:95,961,848-95,961,934	
7q21T_R	GGTCAAGGGAAGACAAACCA		
7q21A1_F	TGGTGAAATGCTGCTCAGTT	chr7:95,963,724-95,963,806	
7q21A1_R	CCTTCGTTTTTCAAGCTGTTG		
7q21B1_F	GAGGGAGCTCTGGAACACTG	chr7:95,972,277-95,972,356	
7q21B1_R	GGAGGAAAACGCAGTAGCAG		
7q21JB1F	TTAGCCCCTTGGATTGACAG	chr7:95,972,977-95,973,061	
7q21JB1R	CCCATGTATGGCTTCCCTTA		

7q21JB2F	GAAGTCTGTGGGCTTGGTTC	chr7:95,975,335-95,975,418	
7q21JB2R	CCAAAGAGTGCCAGAAAACC		
7q21JB3F	CCTGTTGCCCTCCTCATAAT	chr7:95,978,320-95,978,409	
7q21JB3R	TCACCTGCAGTATTTCATGTCC		
7q21C1_F	CTCGTGCTTCATGGAATTGA	chr7:95,980,141-95,980,224	Deleted region
7q21C1_R	TACAGTGCAGCAGGAACAGG		
7q21X_F	TTTGGTTGTGGTTCCTGGAT	chr7:95,982,054-95,982,143	
7q21X_R	CAAAACCACCCATACTCAAAGA		
7q21Y_F	TGCAGTTCAATGGATTTTGC	chr7:95,982,054-95,982,143	
7q21Y_R	CCCATGCCAGCTACAAAAGT		
7q21O_F	CCCTCTGCAAACTAGGTCCA	chr7:96,003,872-96,003,959	
7q21O_R	TGTGTTCAAAAAGCCCCATT		
7q21N_F	GGGTGCAAAATTCGGAGATA	chr7:96,007,547-96,007,635	
7q21N_R	CAATGAAAAGCCTTGCCCTA		
7q21F_F	TCAAAATAACTGATGACGGTGA	chr7:96,037,401-96,037,487	
7q21F_R	TCTATGCCTCTCATGCCACTT		
7q21J_F	TTGCTTTACATTGGTCCATCC	chr7:96,037,795-96,037,874	
7q21J_R	ACAAAGCCAATGCCTGCTAC		
7q21G_F	CACTGCCACACTTTGCTCAT	chr7:96,038,163-96,038,243	
7q21G_R	TAAAACATTTGGGCCTCTGG		
7q21P_F	ACCACATTTTCCCCTACCTTC	chr7:96,111,695-96,111,774	
7q21P_R	GTGATGGAACCAGCATCAAG		
7q21R_F	AGGCATGTGGGAGTTACCAG	chr7:96,118,769-96,118,851	
7q21R_R	TCAGTGGTGTGGCTGTTGTT		
7q21Z_F	GCCTGGCAAAACTGATAGGA	chr7:96,123,569-96,123,653	
7q21Z_R	TGGAGCTGACAATGGAGATG		
7q21Q_F	GCCAACCAGAACAAATGCTT	chr7:96,133,120-96,133,206	
7q21Q_R	GAGGCATGCTTACACATCCA		
7q21D1_F	GTGGACACTGTGCTGAAAGC	chr7:96,136,348-	

7q21D1_R	AGGCAGAGCTGGCAAAATAA	96,136,437	
7q21E1_F	CTTGATGGATCCCAGCTGTC	chr7:96,143,089-96,143,168	
7q21E1_R	AGGAAGCAGTGGAACTGTCC		
7q21F1_F	CCCAAGAAAGGGCCTTAGTC	chr7:96,148,216-96,148,299	
7q21F1_R	CACGTCTTTGTGTGGACCAG		
7q21JB4F	TTTGGAGTCGAACTGGAAGG	chr7:96,149,675-96,149,762	Normal 3' region
7q21JB4R	TGGAGAGACCCAGACACCAT		
7q21U_F	CTGGCTGGGAGGTGATTAAG	chr7:96,151,033-96,151,116	
7q21U_R	TGCCTGGACTACCAAAATCC		
7q21W_F	CTAGCCACAGGAACGGAAAA	chr7:96,198,391-96,198,478	
7q21W_R	AGCCCCTGGTCTGTAGTGAG		
7q21E_F	CTGGATGGTCAGCAACAGAA	chr7:96,503,064-96,503,149	
7q21E_R	ATTTTGGAGAGCCTGCATGT		
Index patient P3			
7q21_F7	GGCTGGTGAACCACAGGTAT	chr7:96,010,420-96,010,502	Normal 5' region
7q21_R7	CAGCAAGGATGAGGAAAAGC		
7q21_F8	TTCTTGGGACTCTGGGTCTG	chr7:96,021,298-96,021,386	
7q21_R8	TCCTTCTTCCTGAGCATCATC		
7q21_F21	CGTCACCAACACTTATTTCCATT	chr7:96,022,149-96,022,238	
7q21_R21	GTGTGCAAACCACAATGACA		
7q21_F22	AAGACGGGTCTGGGCTAACT	chr7:96,023,540-96,023,622	
7q21_R22	GGGTGAGATTCAGCTTGGAA		
7q21_F23	AGGCACCGTATGGACTGGTA	chr7:96,027,097-96,027,178	
7q21_R23	TCAGCAAATTTGTGCTCTGG		
7q21_F24	TGAAAACCAGGTACCCAAGG	chr7:96,031,277-96,031,356	
7q21_R24	TTACTCCTCTCCCTGCCAAA		
7q21_F25	AGAAGTGGGAGAGGCTGTCA	chr7:96,033,377-96,033,462	
7q21_R25	ATGCGCATGTCTTGTGTGTT		
7q21_F26	CAAAGATGGAGGATTTCGTGA	chr7:96,034,465-	

7q21_R26	GAGAAAGACCAGGCCAACTG	96,034,546	
7q21_F9	CCGCATACCTTCAGGGTCTA	chr7:96,036,018-96,036,103	Deleted region
7q21_R9	CAAACTCATTGAGGCATGGA		
7q21_F10	TATTCCCCCAAAGTCTGCTC	chr7:96,171,790-96,171,876	
7q21_R10	CTTCCCCAAAATTGGTCCTT		
7q21_F11	GGGGAGGCAAAGAACAAAAC	chr7:96,181,392-96,181,474	
7q21_R11	CCTTTCCCTGTGGCATTAGA		
7q21_F27	TCTCTTGCCCTTAAGGTTGG	chr7:96,182,414-96,182,495	Normal 3’ region
7q21_R27	ACCCAAGTGGCAGCTCTTC		
7q21_F28	GAAGGCAGTGACCCTGAAAG	chr7:96,183,071-96,183,155	
7q21_R28	TTCCTTATCCCCTGCCTCTT		

Table S2. Sequencing oligonucleotide primers used to establish breakpoints of CNVs and translocations

Primer	Sequence (5’ to 3’, Hg38)	Genomic coordinates	Patient
7q21_F36	GGACCTGAACTCAACACTGGA	chr7:93032409-93032429	P1
7q21_R42	GGATTGACTTGGCGATGC	chr7:97,618,225-97,618,244	
7q21JB3F	CCTGTTGCCCTCCTCATAAT	chr7:95978320-96149762	P2
7q21JB4R	TGGAGAGACCCAGACACCAT		
7q21_F26	CAAAGATGGAGGATTTCGTGA	chr7:96034465-96034485	P3
7q21_R27	ACCCAAGTGGCAGCTCTTC	chr7:96,182,477-96,182,495	
Der7BR_F	CAGGCTCTTTGCACCTGTTA	chr7:96,261,069-96,261,088	P4.1, P4.2, P4.3
Der7BR_R	CCAATTGAGAGGCAAAAGTCA	chr10:79,648,859-79,648,879	
Der10BR_F	CTGAGCACTGATTTTGCTTCA	chr10:79,648,099-79,648,119	
Der10BR_R	GGCCTTCTGCTACAAATCTGA	chr7:96,261,404-96,261,424	

Der7BR_F1	AGGGCTGTCATGAAGAGATTG	chr7:96,510,950-96,510,970	P5
Der7BR_R1	CCCTGTGAGCTTAAAGAGCAA	chr12:97,710,319-97,710,339	
Der7BR_F2	AAAAATCACTGATCCCAGATGAC	chr12:98,132,173-98,132,195	
Der7BR_R2	TGTGAACCAAAAACTCCATCTAA	chr12:78,984,719-78,984,741	
Der7BR_F3	TTCACCATGTGGTTTCATTCA	chr12:79,477,986-79,478,006	
Der7BR_R3	CTGCCTTCCCCTTCACTGTA	chr12:98,132,979-98,132,998	
Der12BR_F1	CCTGTCCTCATGGAGACTACCT	chr12:78,982,951-78,982,972	
Der12BR_R1	CCCAGTTCTGGCTAGTGATTC	chr12:97,703,974-97,703,994	
Der12BR_F2	TGTACACCCATCGTGATAAAAGA	chr12:79,478,782-79,478,804	
Der12BR_R2	TTGATATGGGAGTATGTCCACAA	chr7:96,513,950-96,513,972	

Table S3. Oligonucleotide primers used to perform relative expression analysis

Primer	Sequence (5’ to 3’, Hg38)	GenBank	Target
qTBP_F	CGAATATAATCCCAAGCGGTT	NM_003194.5	Reference gene *TBP*
qTBP_R	TATTTTCTTGCTGCCAGTCTG		
qSYT1_F	GCATAAAATTCCATTGCCACCG	NM_005639.3	*SYT1*
qSYT1_R	ACAAAAGCAGCAGGTCAGGA		
